# Mapeamento de T1 na Insuficiência Cardíaca: Implicações Prognósticas

**DOI:** 10.36660/abc.20210205

**Published:** 2021-05-06

**Affiliations:** 1 Liverpool Heart and Chest Hospital Liverpool Reino Unido Liverpool Heart and Chest Hospital, Liverpool - Reino Unido; 2 National Heart Institute Giza Egito National Heart Institute, Giza - Egito

**Keywords:** Fibrose Endomiocárdica, Insuficiência Cardíaca, Mortalidade, Proteina de Suscetibilidade a Apoptose Celular, Fibroblastos, Ressonância Magnética/métodos

A fibrose miocárdica leva ao comprometimento da função diastólica e sistólica e está associada ao aumento de eventos cardiovasculares adversos maiores. É um correlato estrutural que pode ser encontrado nas diferentes fases da insuficiência cardíaca. Os dois principais tipos de fibrose miocárdica são a fibrose intersticial e a fibrose de substituição. A fibrose intersticial é um processo reversível que ocorre no início do processo da doença como aumento da síntese de colágeno em distribuição microscópica difusa dentro do miocárdio e às vezes por distribuição perivascular localizada. A fibrose de substituição tipicamente ocorre nos estágios mais avançados da doença, após lesão irreversível do miócito ou morte, na qual a apoptose celular desencadeia o aparecimento de fibroblastos e promove deposição macroscópica de tecido fibroso de colágeno no miocárdio.

A ressonância magnética cardíaca (RMC) tem a capacidade de quantificar com precisão os volumes ventriculares e a fração de ejeção, bem como a caracterização não-invasiva do miocárdio. Essas características únicas levaram ao aumento do uso de RMC na avaliação de pacientes com insuficiência cardíaca (IC). A RMC pode detectar a presença e extensão da fibrose de substituição através de imagem de realce tardio com gadolínio e fibrose intersticial difusa por mapeamento de T1 nativo. A fibrose intersticial identificada pelo mapeamento de T1 nativo tem sido utilizada como marcador de atividade da doença,^1^^–^^3^ na estratificação de risco^4^ e no monitoramento do manejo terapêutico em pacientes com IC.^5^

Nesta edição dos Arquivos Brasileiros de Cardiologia, Marques et al.,^6^ relatam a viabilidade da avaliação do mapeamento de T1 nativo em pacientes com IC em um hospital de referência em cardiologia e sua associação com parâmetros estruturais e perfil funcional.^6^ Eles incluíram 134 pacientes com insuficiência cardíaca de diferentes etiologias de um único centro. A maior parte da etiologia da população do estudo foi de pacientes não isquêmicos [n = 95 (70,9%)]. O realce tardio com gadolínio foi observado em 59% (56 pacientes) com cardiomiopatia não-isquêmica e 87% (34 de 39) de pacientes com cardiomiopatia isquêmica. Valores aumentados de T1 nativo do miocárdio foram associados a maiores diâmetros do VE (p = 0,007) e volumes ventriculares (p <0,01). Um valor de T1 significativamente maior foi observado em pacientes com FEVE <35% (p <0,001). Ao comparar os valores de T1 em relação à gravidade da disfunção sistólica, o T1 significativamente maior foi observado na ICFEr do que na ICFEi (p = 0,004) e ICFEp (p <0,001). T1 elevado foi observado em 55,2% dos pacientes com ICFEp (p <0,01).

Além disso, o mapeamento de T1 estava elevado independentemente da etiologia da IC (89,7% em isquêmicos e 81,1% em não-isquêmicos), com um maior valor de T1 observado em pacientes isquêmicos *vs.* não isquêmicos (p = 0,004). Um dado interessante neste estudo, foi o fato de os autores incluíram a cardiomiopatia chagásica. Eles demonstraram que 13 pacientes com cardiomiopatia chagásica com T1 nativo aumentado (1.077,1 ± 61,1ms) estavam associados à redução da FEVE (27,6 ± 16,8%) e aumento dos diâmetros e volumes do VE. Além das diferentes etiologias e da gravidade da insuficiência cardíaca, o tabagismo foi a única comorbidade identificada com valores elevados de T1 estatisticamente significativos (p = 0,032).

Esses achados enfatizam o fato de que o aumento dos valores de mapeamento de T1 nativo apresentaram uma associação direta com os parâmetros tradicionais utilizados para avaliar a gravidade da doença, independentemente da etiologia subjacente. Os autores reconheceram que o tamanho limitado da amostra, outras patologias tais como edema, infiltração, e inflamação podem afetar os valores de T1, bem como a falta de cálculo do volume extracelular. Apesar disso, o mapeamento de T1 nativo oferece um método não invasivo para caracterizar a patologia difusa. Seus achados apoiam o uso do mapeamento de T1 nativo como um biomarcador não invasivo para estratificação de risco na insuficiência cardíaca.
