# Supplementary material for: Mapeamento de T1 na Insuficiência Cardíaca: Implicações Prognósticas
Source: Arq Bras Cardiol. 2021 May 6;116(5):926–7. [Article in Portuguese] doi: 10.36660/abc.20210205 (PMC8121467; doi:10.36660/abc.20210205)
Supplement: Translation

# Translation: T1 Mapping in Heart Failure: Prognostic Implications

**Affiliations:** 1 Liverpool United Kingdom Liverpool Heart and Chest Hospital, Liverpool - United Kingdom; 2 Giza Egypt National Heart Institute, Giza - Egypt

**Keywords:** Endomyocardial Fibrosis, Heart Failure, Mortality, Cellular Apoptose Susceptibility Protein, Fibroblasts, Magnetic Resonance Spectroscopy/methods

Myocardial fibrosis leads to impaired diastolic and systolic function and is associated with increased major adverse cardiovascular events. It is a structural correlate that can be found in the different stages of heart failure. The two main types of myocardial fibrosis are interstitial fibrosis and replacement fibrosis. Interstitial fibrosis is a reversible process that occurs early in the disease process as increased collagen synthesis in diffuse microscopic distribution within the myocardium and sometimes by localized perivascular distribution. Replacement fibrosis typically occurs in the later stages of the disease after irreversible myocyte injury or death, in which cell apoptosis triggers fibroblasts and promotes macroscopic deposition of collagen fibrous tissue in the myocardium.

Cardiac magnetic resonance (CMR) has the capability to accurately quantify ventricular volumes and ejection fraction, as well as the non-invasive characterization of the myocardium. These unique features have led to the increased use of CMR in the assessment of patients with heart failure (HF). CMR can detect the presence and extent of replacement fibrosis through late gadolinium enhancement imaging and diffuse interstitial fibrosis through native T1 mapping. Interstitial fibrosis identified by native T1 mapping has been used as a marker of disease activity,^1^^–^^3^ risk stratification^4^ and monitoring of the therapeutic management in heart failure patients.^5^

In this issue of the Brazilian Archives of Cardiology, Marques et al.^6^ report the feasibility of native T1 mapping assessment in patients with HF in a cardiology referral hospital and its association with structural parameters and functional profile.^6^ They enrolled 134 patients with heart failure of different etiologies from a single center. Most of the study population's etiology comprised non-ischemic patients [n=95 (70.9%)]. Late gadolinium enhancement was observed in 59% (56 patients) with non-ischemic cardiomyopathy and 87% (34 of 39) ischemic cardiomyopathy patients. Increased native myocardial T1 values were associated with larger LV diameters (p = 0.007) and ventricular volumes (p <0.01). A significantly higher T1 value was observed in patients with LVEF <35% (p <0.001). Upon comparing the T1 values in relation to systolic dysfunction severity, significantly higher T1 was observed in HFrEF than in HFmrEF (p = 0.004); and HFpEF (p <0.001). Elevated T1 was observed in 55.2% of patients with HFpEF (p <0.01).

Furthermore, T1 mapping was elevated regardless of the HF etiology (89.7% in ischemic and 81.1% in non-ischemic cases), with a higher T1 value observed in ischemic vs. non-ischemic patients (p = 0.004). Unique to this study, the authors have included Chagas cardiomyopathy. They demonstrated that 13 Chagas cardiomyopathy patients with increased native T1 (1077.1 ± 61.1ms) was associated with reduced LVEF (27.6 ± 16.8%) and increased LV diameters and volumes. In addition to the different etiologies and the severity of heart failure, smoking was the only comorbidity identified with a statistically significant elevated T1 values (p = 0.032).

These findings emphasize that the increased native T1 mapping values had a direct association with traditional parameters used to assess disease severity regardless of the underlying etiology. The authors have acknowledged that the limited sample size, other pathologies such as edema, infiltration, and inflammation may affect T1 values, as well as the lack of extracellular volume calculation. Nonetheless, native T1 mapping offers a noninvasive method to characterize diffuse pathology. Their findings support the use of native T1 mapping as a non-invasive biomarker for risk stratification in heart failure.
